# Persistent Visual Aura following Catheter Ablation in a Patient with WPW Syndrome

**DOI:** 10.1155/2007/274276

**Published:** 2007-08-22

**Authors:** Shinichi Koyama, Mitsuru Kawamura

**Affiliations:** ^1^Department of NeurologyShowa University School of MedicineJapan; ^2^Department of EngineeringChiba UniversityJapan; ^3^The Japan Society for the Promotion of ScienceJapan; ^4^CRESTJSTJapan

**Keywords:** Catheter ablation, migraine, right-to-left shunt, visual aura, Wolff-Parkinson-White (WPW) syndrome

## Abstract

We report a patient who has had persistent visual disturbances since she underwent catheter ablation to treat her Wolff-Parkinson-White (WPW) syndrome. We examined her visual symptoms carefully and quantitatively by means of our newly developed method combining image-processing and psychophysics. We first simulated the patient’s visual symptoms using image-processing techniques. Since the simulation indicated that she would be very sensitive to the edges of the visual stimuli, we evaluated her sensitivity to the edges using psychophysics. The results indicated that she was hypersensitive to the clear-cut edges of the visual stimuli. Her visual symptoms were very similar to those of visual aura of migraine, rather than those of photosensitive epilepsy. Magnetic resonance imaging (MRI) and single photon emission computed tomography (SPECT), electroenchepalogram (EEG), and visual-evoked potentials (VEP) in the patient were normal. No abnormalities in her fundus, visual field, or electroretinogram were found, either. Transesophageal echocardiography with bubble study indicated that she had a preexisting right-to-left shunt. We hypothesize that visual aura of migraine was triggered and made persistent by the catheter ablation in this patient. Although the relationship between migraine, catheter ablation, and right-to-left shunts is unknown, previous studies on the transcatheter closure of patent foramen ovale suggest a possible link between them. Catheter ablation in patients with migraine and preexisting shunts may lead to exacerbations in migraine symptoms.

## 1. Introduction

Although no studies have been done on the relationship between catheter ablation and visual symptoms, at least five patients who experienced catheter ablation have reported on the Internet that they developed visual symptoms and/or migraine after the catheter ablation (see appendix). There may be a link between cardiac catheter procedure and visual symptoms, because patients who underwent transcatheter closure of an atrial shunt also experienced severe visual symptoms immediately aftere the procedure [20,21].

Despite those reports regarding visual symptoms fol-

ISSN 0953-4180/07/$17.00 © 2007 – IOS Press and the authors. All rights reserved lowing cardiac catheter procedure, no detailed studies have been done before. Here we carefully and quantitatively examined visual symptoms of a patient who underwent catheter ablation, by means of our newly developed method combining image-processing and psychophysics. The patient has had persistent visual symptoms continuously for more than four years since she had catheter ablation.

## 2. Methods

### *2.1. Case history*

The patient was a 46-year old Japanese female English teacher who is highly intelligent (Wechsler Adult Intelligence Scale-Revised: verbal IQ = 137, performance IQ = 141, full IQ = 143) and had good memory (Wechsler Memory Scale-Revised: general memory = 111, delayed recall = 115). She started to experience palpitations when she was an elementary school child although it was not detected by electrocardiogram. At age 15, she had a heart attack accompanied with visual disturbances such as seeing moving phosphenes. At age 15 or 16, she started to experience migraine in the back of the head once or twice a month, although she did not experience visual auras at that time. Her migraine was accompanied with pulsating pain and lasted for 2–3 days once it occurred. She was diagnosed with Wolff-Parkinson-White (WPW) syndrome when she had a medical checkup at her college. From the age of 30, she received treatment for her Paroxysmal Supraventricular Tachycardia (PSVT), and she underwent catheter ablation when she was 42. The electrode catheters were inserted from the right subclavian vein and the right femoral vein, and the ablation catheter was inserted from the right femoral artery. The ablation was performed by a transarterial approach, and a transseptal approach [5,9] was not attempted. The Kent bundle was ablated by delivering high-frequency electrical current (35–45W, 55–60$^{\circ}$, 90–120 seconds 5 times) to the lateral left ventricular wall. During the procedures, she lay in the supine position, with the neck and upper limbs in the neutral positions. The ablation was successful, and her PSVT was treated. However, in the middle of the procedure, she developed a strong headache and experienced visual disturbances such as phosphenes, double vision, and visual perseverations. She has since experienced similar visual disturbances. We simulated the patient's visual symptoms using image processing (Fig. 1A): according to the patient, she always sees dazzling spots in the air, similar to the reflection of the water. She also sees afterimages of what she just saw, frequently in inverted color (see the letters, "ABCDEFG" in Fig. 1A), and the afterimages last for a long time. When we showed her the famous Varin figure [17] in which an illusory red square is perceived by normal subjects (Fig. 1B left), she failed to see the visual illusion, and perceived the complimentary color of the color around the edge (Fig. 1B right). The simulation of her visual symptoms (Fig. 1A and B) was done by processing the digital images with the patient and she confirmed that the pictures exactly replicated her visual experiences. She also reported that her migraines worsened in severity and frequency after the ablation, and that her migraines were frequently accompanied with visual aura of scintillating scotoma (Fig. 1C).

She has been prescribed all practicable drugs below at sufficient dose, none of which eased her visual disturbances: [antiepileptics] phenobarbital, sodium valproate, clonazepam, carbamazepine, phenytoin, zonisamide, primidone, acetazolamide, clobazam; [antiheadache drugs] loxoprofensodium, sumatriptan succinate; [antidepressants] imipramine hydrochloride, fluvoxamine maleate, paroxetine hydrochloride hydrate; [antipsychotics] sulpiride, fluphenazine maleate; [other drugs] tofisopam, arotinololhydrochloride, lomerizine hydrochloride, difenidol hydrochloride, and rebamipide. During the 4 years following the ablation, she visited many hospitals to consult ophthalmologists, neurosurgeons, and neurologists on her visual disturbances, but no diagnosis was given until she visited our hospital. No abnormality was found in her magnetic resonance imaging (MRI) and single photon emission computed tomography (SPECT) images. Electroenchepalogram (EEG) (rest, flash, and hyperventilation) showed no abnormalities, either. Visual-evoked potentials (VEP) with a checkerboard pattern were recorded, and her VEP was normal. Ophthalmologists found no abnormalities in her fundus and visual field, and her electroretinogram was normal, too.

### *2.2. Visual tests*

In order to examine her visual symptoms in detail, we conducted a series of visual tests using psychophysics: (1) Brightness perception test: we asked her to arrange gray cards by brightness; (2) Color perception test: Ishihara-test, Panel D-15, and Farnsworth-Maunsell 100 Hue tests; (3) Color-afterimage test [10]; (4) Photosensitivity test similar to those by Wilkins [18] and Hibino and Yamada [7]. In the photosensitivity test,

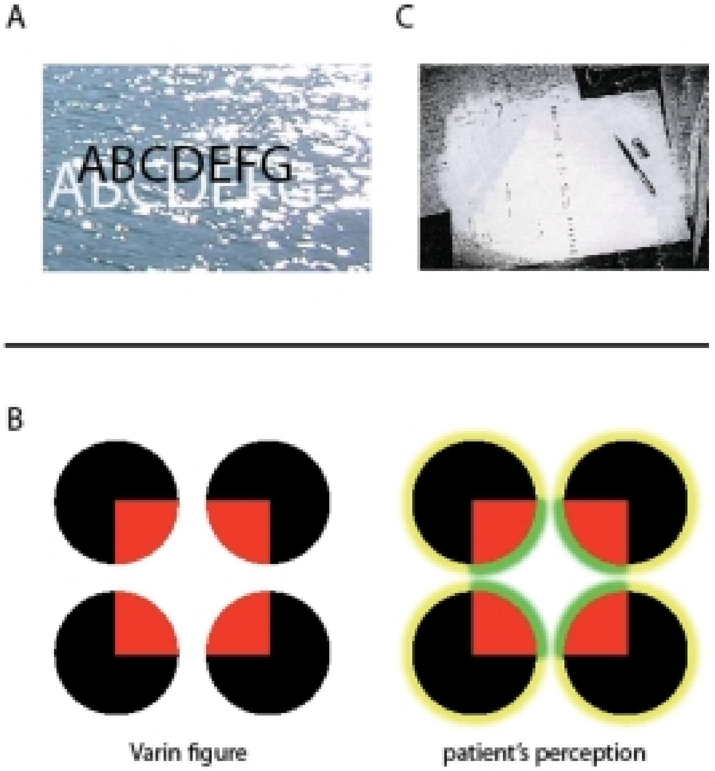

Fig. 1. (A) Simulation of the patient's visual disturbances using image processing. The patient claims that she always sees dazzling spots in the air, similar to the reflection of the water. She also sees afterimages of what she just saw, frequently in the inverted color (see the letters, "ABCDEFG"), and the afterimages last for a long time. The simulation of her visual symptoms was done by processing the digital images with the patient. The patient confirmed that the pictures exactly replicated her visual experiences. (B) Varin figure (left) and the patient's perception of the figure (right). In the left figure, an illusory red square is perceived by normal subjects. Note that the edges of the original Varin figure are surrounded by the "coronas" in the complementary color in the right figure. (C) The patient's drawing of her scintillating scotoma with paint and a photograph. Note that the center of the picture is blank and the white area has a corrugated edge.

the patient and three normal controls viewed a stripe or a grid in each trial, and evaluated the subjective brightness of the stripe or the grid on a 10-point scale, from one (not bright, the brightness of a uniform gray screen) to ten (very bright, as bright as seeing the sun directly). There were two types of stimuli: stimuli with clear-cut edges (square waves) and stimuli with fuzzy edges (sine waves) (Fig. 2A). We used those stimuli in order to see if the subjects were sensitive to the edge or to the pattern. Although both stimuli have patterns such as stripes and grids, clear-cut edges were present only in the square-wave stimuli (Fig. 2A left). Edges were fuzzy in sine-wave stimuli (Figure 2A right). The spatial frequency of the waves (1 cycle per degree) and the average luminance of the display was equal (30 cd/m$^2$) in both stimuli. The size of the grid changed in 3 levels, as shown in Fig. 2B. The above tests were approved by the ethics committee of Showa University School of Medicine and written informed consent was obtained from the patient and the normal controls.

## 3. Results

The patient performed normally on brightness and color perception tests as well as color-afterimage tests. However, the patient was very sensitive to edges in the photosensitivity test. As the number of edges increased in the square-wave stimuli, the patient perceived increasing brightness (Fig. 2B left). With the smallest grids, which she reported as the brightest, tears came from her eyes. On the other hand, the subjective brightness of the normal controls did not increase. In the stimuli with fuzzy edges, the subjective brightness did not increase in the patient or the controls (Fig. 2B right). Taken together, the results of the visual tests indicated that the patient would be extremely sensitive to edges and the results also suggested dysfunction in the lateral inhibition in the visual cortex.

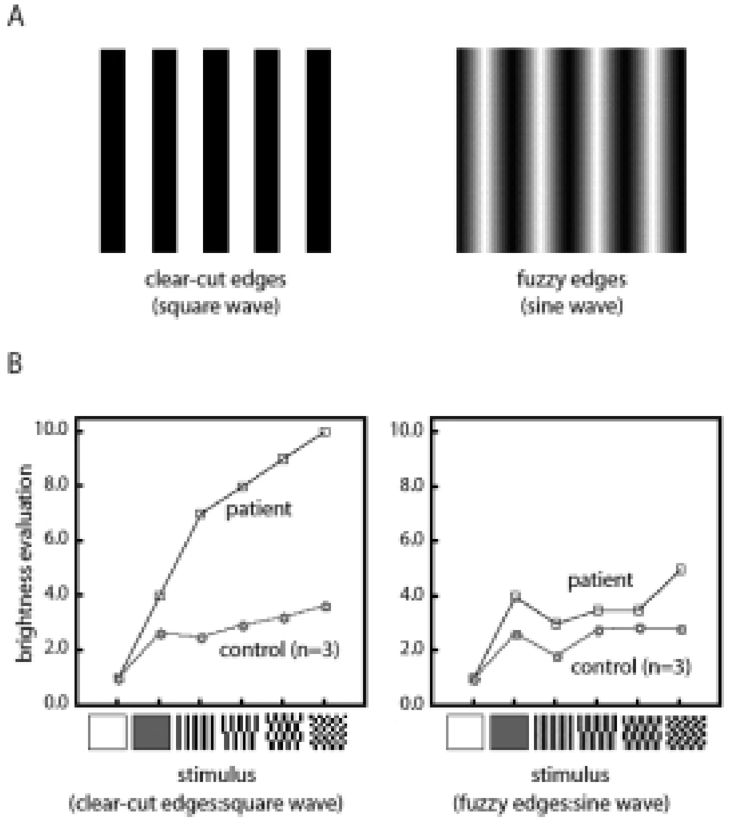

Fig. 2. (A) The stimuli used in the photosensitivity test. Note that the edges are clear-cut in the square-wave stimulus whereas the edges are fuzzy in the sine-wave stimulus. (B) Brightness perception of stripes and grids which consisted of square waves (left) and sine waves (right). The patient was very sensitive to the stripes and grids with clear-cut edges (left) whereas her brightness evaluation was similar to that of normal control in the stripes and grids with fuzzy edges (right).

## 4. Discussion

The results from the visual tests indicated that the patient was hypersensitive to the edges of the visual stimuli. This explains her symptoms (phosphenes, double vision, visual perseveration, and failure to see the Varin figure visual illusion) very well: all the symptoms occur at the edge of the visual stimuli.

The visual symptoms in this patient are very similar to visual aura of migraine. The phenomenon in which edges are perceived as shining is very similar to a type of visual aura called “corona phenomenon [13]”. Phosphenes were typical symptoms of visual aura, too [14]. In addition, the patient frequently experiences scintillating scotoma prior to migraine. Cortical hypersensitivity between the migraine attacks was reported in the studies on VEP [1], transcranial magnetic stimulation (TMS) [2,16], functional MRI [8] and visual psychophysics [22]. Scintillating scotoma prior to migraine was associated with spreading cortical depression prior to the migraine attack [11,12]. The patient’s symptoms were also similar to those of photosensitive epilepsy, although they were different in that the patient perceived the smallest grids brighter than the stripes whereas patients with photosensitive epilepsy are more sensitive to the stripes than grids [18]. In addition, no abnormality was found in her EEG. Therefore, it is most likely that visual symptoms in the patient were visual auras of migraine.

In order to ease her visual symptoms, we advised her to eliminate edges around her by wearing clothes without patterns and using the same color for the wall, curtains, and furniture. We also advised her to wear polarized sunglasses in bluish gray tints, because the polarized lenses filter out diffusively reflecting lights and blue is the complementary color of yellow, i.e., the color of phosphenes that she perceived. The patient reported that those interventions eased her visual disturbances significantly.

Although the relationship between migraine symptoms and catheter ablation is unknown, there may be a link between them. In addition to 5 patients who reported visual disturbances on the Internet, patients who underwent transcatheter closure of patent foramen ovale frequently experienced severe migraine symptoms such as migraine with visual aura, status migrainousus, and visual aura alone, for a few weeks following the closure [20,21]. Those facts suggest that catheter may produce trigger substances for migraine and visual aura (for example, bubbles, microemboli, and vasoactive chemicals [19]) at least for a short period following the surgery.

It was not clear why our patient experienced visual symtoms for more than four years after the catheter ablation. One hypothesis is that the trigger substances could not be filtered out by the lungs because of the right-to-left shunt in this patient. Studies have shown that patients with migraine with visual aura frequently have a right-to-left shunt [4,6,15], and the frequency of visual aura decreases after the closure of right-to-left shunts [3,20]. Indeed, transesophageal echocardiography with bubble study indicated that our patient has a right-to-left shunt. The size and anatomic location of the shunt was unknown, because we could only observe bubbles in the left atrium during the echocardiography. However, her right-to-left shunt is likely to be preexsisting because a transseptal approach [5,9] was not attempted during the catheter ablation in this patient. Although further studies are necessary to examine the relationship between migraine, catheter ablation, and right-to-left shunts, our case study showed that catheter ablation in patients with migraine and preexisting shunts may lead to exacerbations in migraine symptoms.

## Acknowledgements

We thank Tetsuo Furukawa, Haruo Hibino, Makoto Iwata, Juro Kawachi, Yoichi Kobayashi, Masashi Nakajima, Akira Midorikawa, Tatsuya Sato, and Summer Sheremata for helpful comments on this manuscript. We also thank Yuki Kamiya for conducting a transesophageal echocardiography examination. S.K. was supported by the Japan Society for the Promotion of Science. M.K. was supported by CREST, and the Grant-in-Aid for Scientific Research on Priority Areas–System study on higher-order brain functions from MEXT (17022035, 18020027). This study was also supported in part by a Showa University Grant-in-Aid for Innovative Collaborative Research Projects and a Special Research Grant-in-Aid for Development of Characteristic Education from MEXT.

## Appendix

Examples of patients' report on visual symptoms following catheter ablation on the Internet (http://ehealthforum.com/). Note that the data are not peer-reviewed.

Patient 1:

"Yesterday for about 20 minutes I was having visual disturbances. Like when a TV goes fuzzy but in colors out both my eyes. Anyone ever have this happen? ... He (the doctor) told me to go see an eye Dr! I was so mad!"

Patient 2:

"... just wanted to say that after my ablation my eyes did the same thing. I thought I was going blind or something. It was odd as a wierd patch of wiggly colours (only way I can describe it) traveled from one eye to the next and I couldn't see very well."

Patient 3:

"Past history, I had seen stars around then the next day I would have a migraine headache. I've seen the eye specialist, all ok! She suggests CT scan to follow up ... I had been 20 years palpitations free after the WPW ablation."
